# Plant Acyl-CoA:Lysophosphatidylcholine Acyltransferases (LPCATs) Have Different Specificities in Their Forward and Reverse Reactions*

**DOI:** 10.1074/jbc.M113.521815

**Published:** 2013-11-04

**Authors:** Ida Lager, Jenny Lindberg Yilmaz, Xue-Rong Zhou, Katarzyna Jasieniecka, Michael Kazachkov, Peng Wang, Jitao Zou, Randall Weselake, Mark A. Smith, Shen Bayon, John M. Dyer, Jay M. Shockey, Ernst Heinz, Allan Green, Antoni Banas, Sten Stymne

**Affiliations:** From the a Department of Plant Breeding, Swedish University of Agricultural Sciences, S-230 53 Alnarp, Sweden,; b Scandinavian Biotechnology Research AB, S-230 53 Alnarp, Sweden,; c Commonwealth Scientific and Industrial Research Organisation Plant Industry, Canberra, Australian Capital Territory 2601, Australia,; the d Intercollegiate Faculty of Biotechnology of University of Gdansk and Medical University of Gdansk, 80-822 Gdansk, Poland,; the e National Research Council of Canada, Saskatoon, Saskatchewan S7N 0W9, Canada,; the f Department of Biochemistry and Center for Plant Science Innovation, University of Nebraska, Lincoln, Nebraska 68588,; the g Agricultural Lipid Biotechnology Program, Department of Agricultural, Food, and Nutritional Science, University of Alberta, Edmonton, Alberta, T6G 2P5, Canada,; the h Institute of Biological Chemistry, Washington State University, Pullman, Washington 99164,; the i United States Arid-Land Agricultural Research Center, United States Department of Agriculture-Agricultural Research Service, Maricopa, Arizona 85138,; the j Commodity Utilization Research Unit, Southern Regional Research Center, United States Department of Agriculture-Agricultural Research Service, New Orleans, Louisiana 70124, and; the k Biocenter Klein Flottbek and Botanical Garden, University of Hamburg, 22609 Hamburg, Germany

**Keywords:** Arabidopsis, Enzymes, Lipid Metabolism, Phosphatidylcholine, Plant Biochemistry, Lysophosphatidylcholine Acyltransferases, Phosphatidylcholine Metabolism, Plant Lipid Biochemistry, Enzyme

## Abstract

Acyl-CoA:lysophosphatidylcholine acyltransferase (LPCAT) enzymes have central roles in acyl editing of phosphatidylcholine (PC). Plant LPCAT genes were expressed in yeast and characterized biochemically in microsomal preparations of the cells. Specificities for different acyl-CoAs were similar for seven LPCATs from five different species, including species accumulating hydroxylated acyl groups in their seed oil, with a preference for C_18_-unsaturated acyl-CoA and low activity with palmitoyl-CoA and ricinoleoyl (12-hydroxyoctadec-9-enoyl)-CoA. We showed that *Arabidopsis* LPCAT1 and LPCAT2 enzymes catalyzed the acylation and de-acylation of both *sn* positions of PC, with a preference for the *sn*-2 position. When acyl specificities of the *Arabidopsis* LPCATs were measured in the reverse reaction, *sn*-2-bound oleoyl, linoleoyl, and linolenoyl groups from PC were transferred to acyl-CoA to a similar extent. However, a ricinoleoyl group at the *sn*-2-position of PC was removed 4–6-fold faster than an oleoyl group in the reverse reaction, despite poor utilization in the forward reaction. The data presented, taken together with earlier published reports on *in vivo* lipid metabolism, support the hypothesis that plant *LPCAT* enzymes play an important role in regulating the acyl-CoA composition in plant cells by transferring polyunsaturated and hydroxy fatty acids produced on PC directly to the acyl-CoA pool for further metabolism or catabolism.

## Teaser

**Results:** Plant LPCATs were expressed in yeast and biochemically characterized.

**Conclusion:** LPCATs can edit acyl composition of phosphatidylcholine through their combined forward and reverse reactions.

**Significance:** Plant LPCATs play a role in editing both *sn*-positions of PC and remove ricinoleic acid with high selectivity from this lipid.

## Introduction

Plants differ from most other eukaryotes, including animals, in that they synthesize polyunsaturated fatty acids from precursor fatty acids that are esterified to a complex glycerolipid. In the plastids, the preferred lipid substrate for desaturation is monogalactosyldiacylglycerol, and in the cytosol it is phosphatidylcholine (PC)^3^ (1). The polyunsaturated fatty acids formed on PC are distributed in all cytosolic lipid classes in the plant cell, including the triacylglycerols (TAGs) that accumulate in massive amounts in oil-storing tissues. This necessitates an efficient mechanisms whereby the monounsaturated fatty acid, *i.e.* oleic acid (18:1*^cis^*^Δ9^), is channeled into PC for further desaturation, and the resulting polyunsaturated fatty acids, mainly linoleic (18:2*^cis^*^Δ9,12^) and α-linolenic (18:3*^cis^*^Δ9,12,15^) acids, are channeled to other lipids. It has been shown that PC also is the substrate for the biosynthesis of a number of unusual fatty acids in plants, such as hydroxy-, epoxy-, acetylenic, and conjugated fatty acids. These fatty acids are formed by the catalytic action of Δ12-desaturase-like (FAD2) enzymes (2). Some plants accumulate unusual fatty acids to a very high percentage in the TAGs while maintaining very low levels in seed PC, their site of synthesis (3, 4). Thus, in the case of these unusual fatty acids, the cell does not only need an efficient mechanism of channeling them from PC to TAG but also the mechanisms that remove them from PC with high selectivity.

In animal cells, the turnover of acyl groups in PC is believed to be accomplished by phospholipase-catalyzed release of free fatty acid forming lysophosphatidylcholine (LPC). LPC is subsequently reacylated utilizing acyl-CoA via the catalytic action of an acyl-CoA:lysophosphatidylcholine acyltransferase (LPCAT, EC 2.3.1.23) in the so-called Lands cycle (5). This cycle has also been suggested to be involved in the channeling of ricinoleic acid (12-hydroxyoctadec-9-enoic acid), vernolic acid (12-epoxyoctadec-9-enoic acid), and crepenynic acid from PC to TAG in plants (6–8).

It has been shown that there is a rapid interconversion between diacylglycerols (DAG) and PC during TAG synthesis in some oil seeds (9, 10). This interconversion was originally suggested to be catalyzed by the reverse and forward reaction of the CDP-choline:diacylglycerol cholinephosphotransferase (11), but recently a novel enzyme was identified, phosphatidylcholine:diacylglycerol cholinephosphotransferase (PDCT) that could efficiently carry out this reaction in *Arabidopsis* seeds (12). Other routes of transfer of acyl groups from PC to TAGs and steryl esters in plants are catalyzed by phospholipid:diacylglycerol acyltransferase (PDAT) and phospholipid:sterol acyltransferase, respectively (13, 14).

An acyl exchange between PC and acyl-CoA was demonstrated in microsomal preparations from developing soybean over 30 years ago (15). A few years later, in experiments with microsomal preparations from developing safflower seeds, it was suggested that this exchange was catalyzed by the combined forward and reverse reaction of an LPCAT enzyme (16). However, it was not until recently that the plant *LPCAT* genes were cloned (17), and this hypothesis could be more rigorously tested. In the work reported here, we confirm that plant LPCAT enzymes can operate in a reversible fashion *in vitro*. Our experiments also support the hypothesis that LPCATs play a significant role *in vivo* in the exchange of acyl groups between acyl-CoA and PC pools. We also show that, unexpectedly, LPCAT can have an important role in specifically removing unusual fatty acids formed on PC. Furthermore, we report on the positional specificities, acyl specificities, and selectivities of plant LPCAT enzymes.

## EXPERIMENTAL PROCEDURES

### 

#### 

##### Chemicals

[1-^14^C]18:1 and [1-^14^C]palmitoleic acid (16:0) were purchased from PerkinElmer Life Sciences. [1-^14^C]Ricinoleic acid was obtained from American Radiochemicals. Nonradioactive fatty acids, free CoA, *sn*-1–18:1-LPC and *sn*-1–16:0-LPC, fatty acid methyl ester, and fatty alcohol standards were obtained from Larodan (Malmö, Sweden). Acyl-CoAs were prepared according to the method described by Sánchez *et al.* (18). Molecular species of *sn*-1–16:0-*sn*-2-[^14^C]acyl-PC and [^14^C]18:1-LPC were prepared by acylation of the trifluoroacetic anhydride of the radioactive fatty acid to *sn*-1–16:0-LPC and glycero-*sn*-3-phosphorylcholine (Sigma), respectively, according to Ref. 19. *sn*-1–16:0-*sn*-2-[^14^C]ricinoleoyl-PC was prepared by incubating microsomes from yeast expressing the AtLPCAT2 with [^14^C]ricinoleoyl-CoA as in assays for forward reaction of LPCATs as described under “Enzyme Assays” for 30 min with nonradioactive 16:0-LPC and [^14^C]ricinoleoyl-CoA. GC analysis of methylated fatty acids (see under “Lipid Extraction, Separation, and Analysis”) from the purified PC showed that 80% of the PC species consisted of *sn*-1–16:0-*sn*-2-ricinoleoyl-PC. l-*O*-9-*cis*-Octadecenyl-*sn*-glycero-3-phosphocholine (*sn*-1-OGPC) and 2-*O*-(9-*cis*-octadecenyl)-*sn*-glycero-3-phosphocholine (*sn*-2-OGPC) were synthesized as described previously (20). Ricinoleoyl-LPC was produced by phospholipase A_2_ (from *Naja naja*, Sigma) treatment of di-ricinoleoyl-PC (kindly provided by ENI/Metapontum Agrobios, Metaponto, Italy) according to Ref. 6.

##### Yeast Strains and Plasmids

The *Saccharomyces cerevisiae* haploid knock-out mutant of *ALE1*:(BY4741; *Mata*; his3Δ1; leu2Δ0; lys2Δ0; ura3Δ0; YOR175c::kanMX4) was used as host strain for the expression of the LPCAT enzymes. Two variants of the plasmid pYES (Invitrogen) were used for expressing the LPCATs in yeast under the control of the GAL1 promoter. AtLPCAT1 (At1g12640), AtLPCAT2 (At1g63050), LfLPCAT2, McLPCAT, and AfLPCAT were expressed in pYES2, whereas CtLPCAT, RcLPCAT, BpLPCATs, and HbLPCATs were expressed in pYES-DEST52. The *LPCAT* sequences were confirmed by sequencing and introduced into the yeast strain *ale1*. An empty vector pYES2 was used as a control in the experiments.

##### Yeast Cultivation and Microsomal Preparations

Recombinant yeast cells were grown at 30 °C in synthetic uracil drop-out medium containing 2% galactose. After 24 h, yeast cells were harvested, washed with 20 mm Tris-HCl, pH 7.9, and resuspended in extraction buffer (20 mm Tris-HCl, pH 7.9, 10 mm MgCl_2_, 1 mm EDTA, 5% (v/v) glycerol, 1 mm DTT, 0.3 m ammonium sulfate) containing protease inhibitor (Complete, Roche Applied Science). The cells were disrupted by homogenization with 0.5-mm zirconia/silica beads using a Mini Beadbeater-8 (Biospec Products). The homogenates were centrifuged at 1,500 × *g*, and supernatants were transferred to new tubes, diluted with extraction buffer, and centrifuged 100,000 × *g* for 2 h at 4 °C. The pellets were resuspended in 0.1 m potassium phosphate, pH 7.2, and these extracts, subsequently referred to as microsomal membranes or microsomes, were stored at −80 °C.

##### Enzyme Assays

The enzyme assays were performed with microsomal membranes prepared from *ale1* yeast expressing an *LPCAT* gene. Control microsomes were prepared from *ale1* yeast transformed with empty vector. Assays for measuring the forward reaction of the LPCATs contained 10 nmol of acyl-acceptor (18:1-LPC/1-OGPC/2-OGPC), 5 nmol of acyl-CoA (if single substrate assays, otherwise 10 nmol of total acyl-CoA), 0.1 m potassium phosphate buffer, pH 7.2, in a total volume of 50 μl. ^14^C-Labeled fatty acids were either in the LPC or in the acyl-CoA substrate, depending on the experiment, as indicated in the table or figure legends. The amount of microsomes optimized to assay under linear conditions varied from 0.1 to 0.8 μg of microsomal protein. Acylation of positional isomers of OGPC and LPC was performed with 0.5 and 5 μg of microsomal protein in assays without and with fatty acid-free bovine serum albumin (BSA) (20 mg/ml). The reaction time was 4 min at 30 °C. LPCAT-specific activity measured in different microsomal preparations from yeast with the same *LPCAT* gene expressed could differ by up to 50%. Assays measuring the reverse reaction and acyl exchange contained 43 μg of microsomal proteins, 200 nmol of free CoA, and 1 mg of BSA in 0.1 m potassium phosphate buffer, pH 7.2, in final volume of 100 μl. In some of the assays, dithionitrobenzoic acid (DTNB) was added at concentrations as stated in the figure texts. When [^14^C]PC species were used as substrate for the reverse reaction, *sn*-1–16:0-*sn*-2[^14^C]acyl-PC (9 nmol or, in case of mixed substrate, 4.5 nmol of each PC species) was added to freeze-dried microsomes in benzene according to the method described previously (21). Phosphate buffer, pH 7.2, was then added together with BSA, CoA, and 10 nmol of 18:1-CoA, and the assays were incubated 60 min at 30 °C.

To investigate if pre-existing LPC in the microsomal membranes would influence the results of the assays, we measured the LPC concentration in two microsomal preparations with LPCAT expressed (AtLPCAT1 and RcLPCAT) in triplicate samples. The amount of LPC varied in the two preparations between 23 and 58 nmol per mg of microsomal protein. In assays with LPC added, the endogenous LPC constituted about 0.06 to 3% of added LPC and would thus not significantly influence the assay results. To estimate the amount of endogenous acyl-CoA, we incubated microsomes (5 μg of microsomal protein) for 4 min with [^14^C]18:1-LPC without addition of acyl-CoA and measured the incorporation into PC. Because yeast microsomes contain lysophospholipases that also have lysophospholipid transacylase activity and thus are able to produce PC (22), we incubated microsomes with two different LPCATs expressed (AtLPCAT1 and RcLPCAT) and compared the results with incubations of microsomes of *ale1* strain transformed with empty vector. Radioactive PC in the latter incubation was regarded to be formed only by the endogenous lysophospholipase/transacylase and the former by the combined action of endogenous phospholipase and LPCAT utilizing endogenous acyl-CoA. The vector control and LPCAT-expressing membranes produced 0.15 ± 0.03 nmol of PC and 0.2 ± 0.16 nmol per assay, respectively. It can be estimated from these figures that the dilution of added acyl-CoA by endogenous acyl-CoA in the assays would be in the range of 0.05–1% and would thus not significantly influence the obtained results.

##### Lipid Extraction, Separation, and Analysis

The microsomal assays were terminated by addition of 170 μl (in case of 50-μl assay volume) or 100 μl (in case of 100-μl assays) of 0.15 m acetic acid and 500 μl of chloroform/methanol (1:1, v/v) and vortexed. After centrifugation, the lower (chloroform) layer was removed, and an aliquot was taken to liquid scintillation counting of the radioactivity. The rest of the lower phase was applied on silica thin layer chromatography (TLC) plate (Silica 60, Merck), and the plate was developed in polar solvent, chloroform/methanol/acetic acid/water (90:15:10:3, v/v/v.). Radioactive spots were visualized and identified by *R_f_* values of authentic standards, and the relative amount of radioactivity in each spot was determined by InstantImager (Packard Instrument Co.) electronic autoradiograph. Absolute amounts of radioactivity in each spot were calculated from the total amount of radioactivity in the chloroform phase as determined by liquid scintillation. In assays with positional isomers of OGPC and LPC and measuring acyl substrate selectivity, 18 assays were pooled before TLC. PC was recovered from the plate and methylated. Methyl esters were analyzed either by GC analysis as described under “Fatty Acid Analysis” (in case of assays with nonradioactive acyl-CoAs) or by argentation TLC (hexane/diethyl ether/acetic acid; 70:30:1, v/v/v) and subsequent determination of distribution of radioactivity between acyl groups with InstantImager.

When assays were done with BSA (reverse reaction), all the acyl-CoA partitioned in the upper phase with a majority bound to denatured BSA protein (23). After removing the chloroform phase, the upper phase was thoroughly washed three times with 2.5 ml of chloroform. 0.5 ml of 4 m KOH was added to the washed upper phase, and the solution was heated at 90 °C for 15 min to hydrolyze the acyl-CoA. After acidification with HCl, the free fatty acids were extracted into chloroform by adding chloroform/methanol according to the proportions devised by Bligh and Dyer (24). The amount of ^14^C-labeled free fatty acids in the chloroform phase was determined by liquid scintillation and was regarded as the total radioactivity in the acyl-CoA fraction. No significant radioactivity was found in this phase in incubation with control microsomes. In assays with mixed [^14^C]18:1-PC and [^14^C]ricinoleoyl-PC species, ^14^C-labeled free fatty acids were separated on silica TLC plates in PC hexane/diethyl ether/acetic acid (50:50:1, v/v/v). In assays with mixed [^14^C]18:1-PC, [^14^C]18:2-PC, and [^14^C]18:3-PC, the fatty acids were methylated (see below) and separated on argentation TLC plates in hexane/diethyl ether/acetic acid (85:15:1, v/v/v) The relative amount of radioactivity in each fatty acids was quantified by electronic autoradiography using InstantImager.

Analysis of radioactivities at the different *sn* positions of [^14^C]PC isolated after assays with [^14^C]acyl-CoA under conditions promoting acyl exchange was performed by phospholipase A_2_ (from *N. naja*, Sigma) hydrolysis according to Ref. 6. The products from hydrolysis were separated in the polar TLC system as described above, and the relative radioactivity in [^14^C]LPC and [^14^C]free fatty acids was determined by Instant Imager.

##### Fatty Acid Analysis

Free fatty acids and acyl groups of acyl-CoAs and lipids were analyzed and quantified by GC analysis after conversion to corresponding methyl esters by heating in 2 ml of 2% H_2_SO_4_ in water-free methanol in capped tubes at 90 °C for 30 min. The methyl esters were then extracted into hexane by adding hexane (2 ml) and water (2 ml). GC analysis of fatty acid methyl esters was performed on a CP-wax 58 (FFAP-CB) column using a Shimadzu gas chromatograph. Helium was used as carrier gas at a column flow rate of 7.7 ml/min. The injection and detector temperatures were 230 and 270 °C, respectively. Initial temperature was set at 100 °C, and the temperature was raised at a rate of 15 °C/min up to 160 °C, and then 10 °C/min up to 250 °C, and held at 250 °C for 20 min. The identification of fatty acid methyl esters were performed by comparing the retention times with authentic standards. Quantification of fatty acid methyl esters was done by addition of heptadecanoic acid methyl esters as internal standard.

## RESULTS

### 

#### 

##### Reversibility of LPCAT-catalyzed Reactions

**FIGURE 1.**
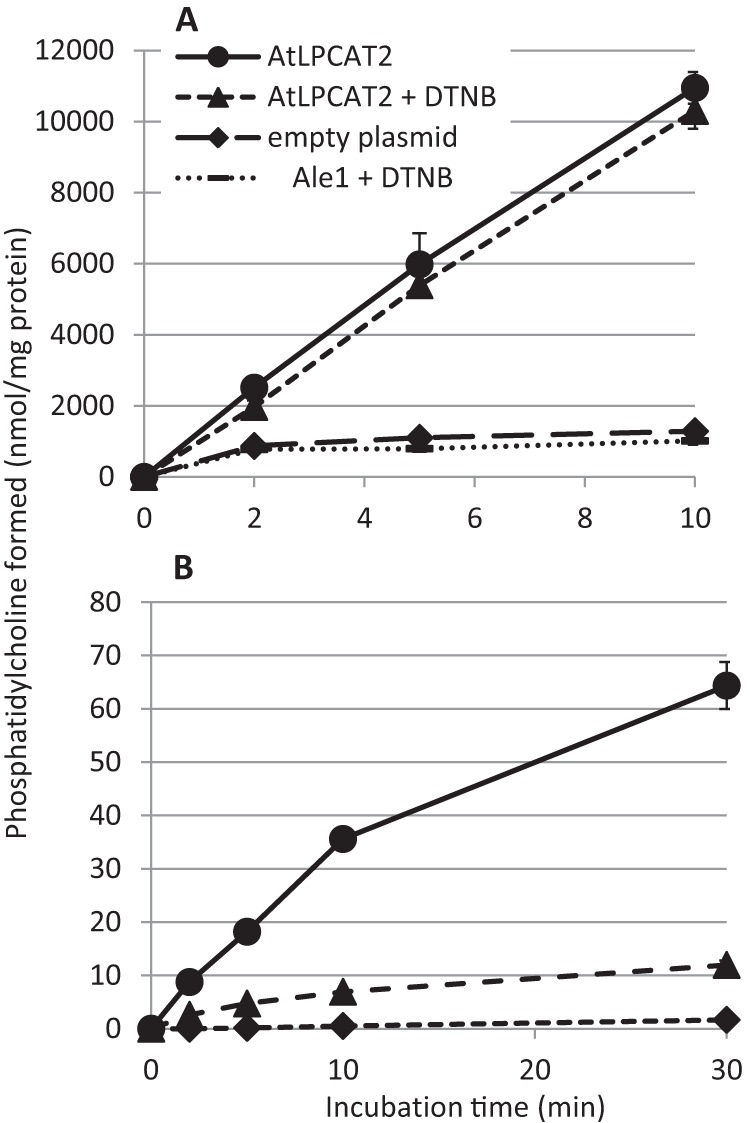
**Time course of [^14^C]18:1 incorporation from [^14^C]18:1-CoA into PC in microsomal preparations of the *ale1* yeast strain expressing AtLPCAT2.** *A*, incorporation of [^14^C]18:1 into PC in the presence of added LPC (forward reaction). *B,* incorporation of [^14^C]18:1 into PC in the absence of LPC with added BSA and free CoA (incorporation via acyl exchange). Incubation conditions as assays for forward reaction (*A*) and reverse reaction without addition of LPC (*B*) (see “Experimental Procedures”) are shown. The DTNB concentration was 5 mm. Assays were performed in duplicate, and *error bars* are given ± S.D.

The forward reaction of LPCAT, *i.e.* the acylation of LPC to form PC by the use of acyl-CoA, involves the breaking of a thioester bond and forming an oxygen ester. This is a strongly exothermic reaction, and thus equilibrium of the LPCAT reaction favors the formation of PC. To have a relevant physiological activity in the cell in the reverse reaction, the catalytic activity of the LPCAT enzyme or the amount of enzyme present has to be very high. In addition, the pool sizes of the substrates for the forward reaction have to be much smaller than for the reverse reaction. We expressed *Arabidopsis* LPCAT2 (AtLPCAT2) in a yeast strain bearing a mutation in the endogenous *LPCAT* gene (*ALE1*) and measured LPCAT activity in the forward reaction in microsomal preparations by adding LPC and [^14^C]18:1-CoA under optimized conditions (Fig. 1*A*). The reaction was linear for at least 10 min with a specific activity of about 1 μmol/min/mg of protein. LPCAT activity in incubations with microsomes from *ale1* yeast transformed with empty vector as control was less than one-tenth of the activity in microsomes with the expressed LPCAT2 (Fig. 1*A*).

**FIGURE 2.**
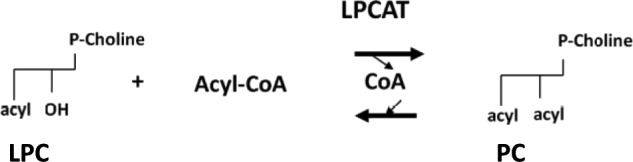
**Reactions catalyzed by LPCAT.** The acylation of acyl groups from PC to CoA is favored by high concentrations of PC and CoA and low concentrations of LPC and acyl-CoA. By adding free CoA and binding acyl-CoA to BSA in the microsomal assays, the equilibrium of the reaction is shifted toward the reverse reaction. The LPC concentration will be maintained low in the assays by the reacylation of the LPC formed by the backward reaction by the forward reaction of the LPCAT enzyme.

DTNB is an effective scavenger of sulfhydryl groups and thus of free CoA. Because free CoA is necessary for the reverse reaction of LPCAT, DTNB is expected to block this reaction. DTNB additions up to 5 mm had only a slight inhibitory effect on the forward reaction of LPCAT (Fig. 1*A*). Previous *in vitro* experiments showed that addition of BSA is essential for the reversibility of LPCAT and that BSA can be replaced by a *Brassica napus* acyl-CoA-binding protein (ACBP) (16, 25). However, addition of excess of BSA strongly inhibits the forward reaction (16). The most plausible explanation for these effects is that acyl-CoA bound to BSA or ACBP cannot be utilized by the LPCAT enzyme. Therefore, the presence of BSA or ACBP effectively lowers the pool size of acyl-CoA available for the forward reaction of the enzyme and thus favors the reverse reaction. To assess the rate of the reverse reaction of AtLPCAT2 under conditions that would favor this direction, we incubated the microsomes from yeast expressing AtLPCAT2 with [^14^C]18:1-CoA in the presence of BSA and free CoA but in the absence of added LPC (Fig. 1*B*). The rationale behind this experiment is that any endogenous LPC will effectively be acylated in the forward reaction catalyzed by LPCAT. Further generation of LPC can therefore only be achieved by the reverse reaction of LPCAT or by endogenous yeast phospholipases. The generated LPC will be rapidly acylated by the forward reaction of LPCAT. In this way, incorporation of radioactivity from [^14^C]18:1-CoA into PC will continue until an equilibrium between acyl groups in PC and acyl-CoA is achieved or the acyl-CoA is depleted due to hydrolysis by endogenous yeast enzymes (Fig. 2).

The incorporation of [^14^C]18:1-CoA into PC under these assay conditions was essentially linear for the first 10 min with an incorporation rate of about 3.5 nmol/min/mg of protein and continued at a reduced rate up to at least 30 min (Fig. 1*B*). Addition of DTNB caused about 80% inhibition of the incorporation measured after 10 min of incubation; the incorporation rate was then 0.7 nmol/min/mg of protein (Fig. 1*B*). Because the acylation rate in the presence of DTNB is likely to represent acylation of pre-existing LPC and formation of LPC by endogenous enzymes (*i.e.* phospholipases), the actual acyl exchange rate can be calculated as 3.5–0.7 = 2.8 nmol/min/mg of protein. Although this represents only 0.3% of the optimal forward rate (Fig. 1*A*), it is in the same magnitude of rate as measured for the forward reaction of other microsomal acyl-CoA acyltransferases expressed in yeast (26, 27). It should be noted that incorporation of radioactivity into PC in control microsomes prepared from yeast carrying the empty plasmid was just above the detection limit (Fig. 1*B*), demonstrating that no enzyme other than AtLPCAT2 was present that could efficiently catalyze the incorporation of acyl groups into PC by acyl exchange.

##### Acyl Specificities of LPCATs in Forward and Reverse Reaction

An acyltransferase may be expected to have the same specificities in the reverse reaction as it has in the forward reaction. However, acyl-CoA (forward reaction) and PC (reverse reaction) are acyl donors with significant differences in structure and acyl bond stability, which may affect their presentation, affinity, and turnover by the LPCAT enzyme. In the reverse reaction, the membrane-bound enzyme is embedded in its acyl donors, which represent components of this membrane. Therefore, the accessibility/reactivity of the surrounding PC molecular species could be quite different compared with the acyl-CoA species presented to the enzyme.

We first studied the acyl specificities in the forward reaction of AtLPCAT1 and AtLPCAT2 in microsomes of the *ale1* yeast expressing the genes encoding these enzymes. The specificities were compared with the selectivity by presenting the enzymes to a mixture of acyl-CoAs. We also included ricinoleoyl-CoA in our assays even though ricinoleic acid is normally not produced in *Arabidopsis*. The reason for this was to determine the activity of *Arabidopsis* LPCATs for this unusual fatty acid as it is formed on PC, and production has previously been engineered in *Arabidopsis* by the ectopic expression of a Δ12-hydroxylase gene (28–31).

**FIGURE 3.**
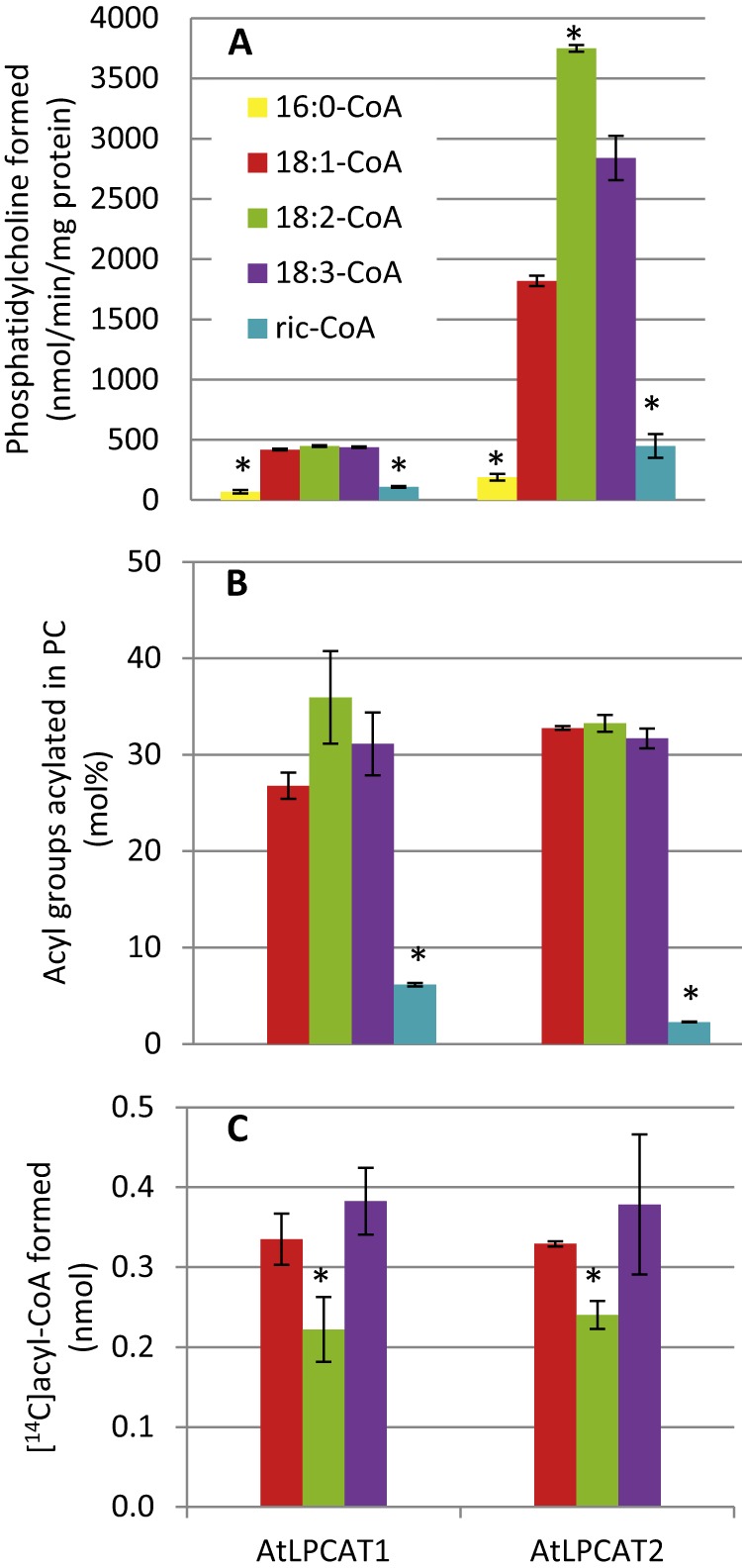
**Acyl substrate specificities and selectivities of AtLPCAT1 and AtLPCAT2.** *A,* acyl specificities in the forward reaction. Acyl-CoA substrates were provided as single substrates with added [^14^C]18:1-LPC. *B,* acyl selectivities in the forward reaction. Acyl-CoA substrates (18:1-, 18:2-, 18:3-, and ricinoleoyl-CoA) were provided as an equimolar mixture with [^14^C]18:1-LPC. *C,* acyl specificities in the reverse reaction. *sn*-2-[^14^C]18:1-PC, *sn*-2-[^14^C]18:2-PC, and *sn*-2-[^14^C]18:3-PC substrates were provided as single substrates. The different [^14^C] acyl groups trapped in the acyl-CoA fraction (see “Experimental Procedures”) were regarded as a result of the LPCAT reverse reaction. The data are from duplicate (*A* and *B*) and triplicate (*C*) assays ± S.D. A significant difference (*t* test, *p* < 0.05) to 18:1-CoA is denoted with an *asterisk. Ric-CoA*, ricinoleoyl-CoA.

Both LPCATs had low activities with palmitoyl- and ricinoleoyl-CoA when these acyl-CoAs were presented as single substrates. Thioesters of the common unsaturated C18 fatty acids, 18:1, 18:2, and 18:3, were acylated at about the same rate by the catalytic action of LPCAT1, whereas LPCAT2 showed relatively lower activity with 18:1-CoA than with 18:2-CoA (Fig. 3*A*). The specific activity of LPCAT1 was 15–20% of the LPCAT2 activity, but differences in actual expression levels of both enzymes were not determined. It should also be noted that the specific activity of the same LPCAT could vary up to 50% between different microsomal preparations (see under “Experimental Procedures”). We then measured relative incorporation from an equimolar mixture of 18:1-CoA, 18:2-CoA, 18:3-CoA, and ricinoleoyl-CoA (Fig. 3*B*). The relative incorporation of ricinoleoyl groups into PC was small in assays with either enzyme. Both enzymes catalyzed the incorporation of all the other acyl groups in roughly the same proportions.

To measure the acyl specificity in the reverse reaction, we presented *sn*-2-^14^C-labeled 18:1-, 18:2-, and 18:3-PC to the membrane as single substrates and assayed under conditions favoring the reverse reaction. In these assays, we added an unlabeled 18:1-CoA to trap the [^14^C]acyl-CoA formed from [^14^C]PC in the acyl-CoA pool. Thus, any LPC formed by the reverse reaction of LPCAT would be acylated mainly with unlabeled 18:1-CoA, whereas the acyl groups removed from PC would be mainly found as acyl-CoA. We then determined the [^14^C]acyl amount and distribution in the acyl-CoA fraction. Both enzymes transferred 18:1 and 18:3 acyl groups from PC to acyl-CoA at similar rates, with 18:2 being utilized at a somewhat lower rate (Fig. 3*C*). It can be noted that the amounts of acyl groups found in the acyl-CoA fraction were virtually the same for both LPCATs, although the forward activity of LPCAT2 was about five times higher than that of LPCAT1 with these acyl groups (Fig. 3*A*).

##### Positional Specificities of the Arabidopsis LPCATs

To determine the positional specificities of the *Arabidopsis* LPCAT, we used ether analogs of 18:1-LPC, l-*O*-9-*cis*-octadecenyl-*sn*-glycero-3-phosphocholine (*sn*-1-OGPC), and 2-*O*-(9-*cis*-octadecenyl)-*sn*-glycero-3-phosphocholine (*sn*-2-OGPC), as acyl acceptors. This was done because *sn*-2-LPC is unstable, and acyl groups will rapidly migrate to the *sn*-1 position. It was previously shown that microsomal preparations of developing sunflower seeds can acylate both these ether analogs with acyl-CoA (20). Similar specificities and rates of acylation as with *sn*-1-LPC were found with the *sn*-1-OGPC, with 16:0-CoA being a very poor substrate (Table 1). Although both *sn*-1 and *sn*-2 ether homologs of LPC were acylated by the catalytic action of both LPCAT1 and LPCAT2, the rate of acylation was 6–7 times higher with the *sn*-1 substrate than with *sn*-2 substrate when 18:1-CoA was used as an acyl donor (Table 1). With 16:0-CoA as a substrate, the acylation rate with *sn*-1-OGPC was about 3–5-fold that of the *sn*-2-OGPC. Addition of BSA to the assays with [^14^C]18:1-CoA led to a 20–30-fold decrease in acylation rate of *sn*-1-LPC and *sn*-1-OGPC but only to a 5-fold decrease with *sn*-2-OGPC. BSA had little effect on the acylation rate of 16:0 with all the acyl acceptors, giving an acylation rate of *sn*-1-OGPC that was about two times that of the *sn*-2-OGPC (Table 1).

**TABLE 1:**
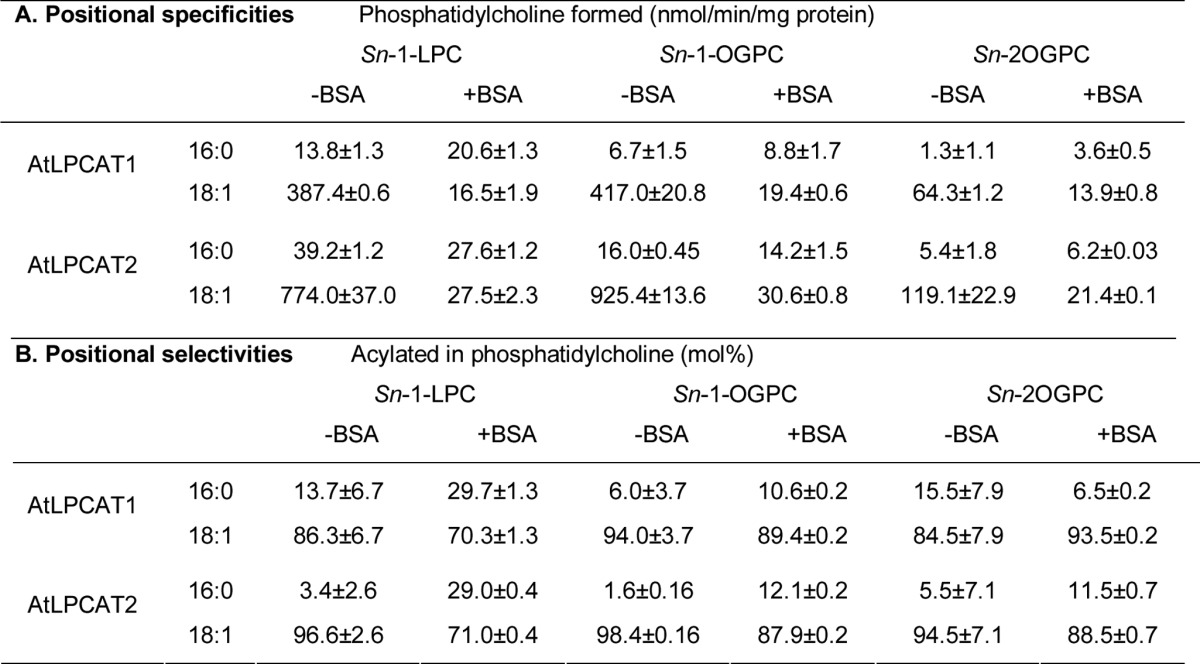
**Positional specificities (A) and selectivities (B) of the *Arabidopsis* LPCATs** A, shown are assays with single [^14^C]acyl-CoA substrates. B, shown are assays with an equimolar mixture of [^14^C]16:0-CoA and [^14^C]18:1-CoA. The abbreviations used are as follows: *sn*-1-OGPC, ether analog of *sn*-1-LPC; *sn*-2-OGPC, *sn*-2 ether analog of *sn*-2-LPC; LPC, *sn*-1-18:1-LPC. The assays were done with incubation conditions for the forward reaction (see under “Experimental Procedures”). The data are from duplicates ± S.D.

Because saturated fatty acids, like 16:0, are nearly exclusively found at the *sn*-1 position of plant phospholipids, we investigated if the LPCAT selected 16:0-CoA at a higher degree in the acylation of the *sn*-1 position when an equimolar mixture of 16:0-CoA and 18:1-CoA was presented to the enzyme. However, 16:0 was strongly selected against in acylation of the *sn*-2 ether analog of LPC by both AtLPCATs, regardless if BSA was added or not in the assays (Table 1).

**FIGURE 4.**
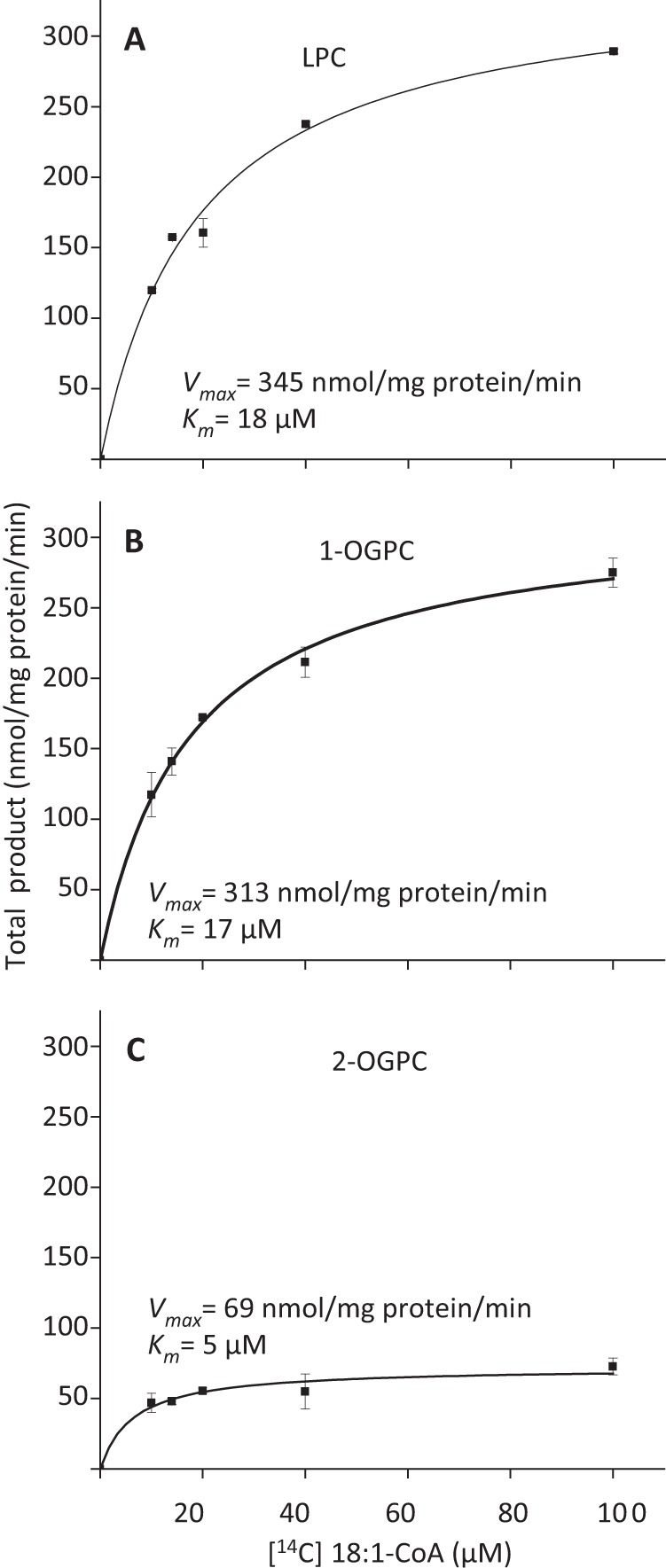
**Kinetics of acylation of different acyl acceptors using [^14^C]18:1-CoA as an acyl donor, catalyzed by AtLPCAT1.** *LPC*, *sn*-1–18:1-LPC; 1*-OGPC*, *sn*-1-*O*-(9-*cis*-octadecenyl)-*sn*-glycero-3-phosphocholine; *2-OGPC*, *sn*-2-*O*-(9-*cis*-octadecenyl)-*sn*-glycero-3-phosphocholine. Assays were performed as described for the forward reaction under “Experimental Procedures” with acyl-CoA concentrations as indicated in the figure. The results presented are from duplicate assays ± S.D.

To kinetically explain the effects of BSA addition on the acylation of the positional OGPC isomers with 18:1-CoA as acyl donor (Table 1), apparent *K_m_* values for 18:1-CoA were determined for LPCAT1 using LPC, 1-OGPC and 2-OGPC as acyl acceptors. The *V*_max_ and *K_m_* values for 18:1 using LPC or 1-OGPC were virtually identical (Fig. 4). However, the *K_m_* value for 18:1-CoA in the acylation of *sn*-2-OGPC was 4.5 times lower than measured for the *sn*-1 isomer (Fig. 4). Thus, the higher ratio of *sn*-1 to *sn*-2 acylation of OGPC seen when BSA was added (Table 1) can most easily be explained by the assumption that acyl-CoA bound to BSA is unavailable for the enzyme. A decrease in the acyl-CoA substrate concentration will affect the rate of acylation of *sn*-2-OGPC less than the rate of *sn*-1-OGPC acylation. Attempts to determine kinetic parameters for acyl-CoA with LPCAT2 failed because we could not obtain reaction conditions that followed the Michaelis-Menten kinetics (data not shown).

**FIGURE 5.**
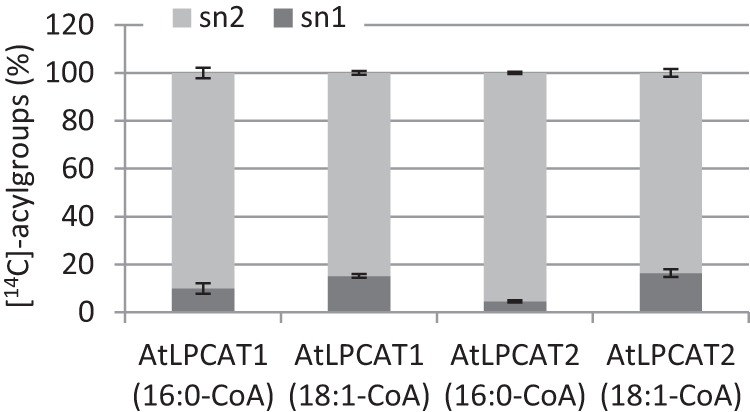
**Relative incorporation of [^14^C]16:0 and [^14^C]18:1 from acyl-CoA into the different *sn* positions of PC by AtLPCAT1 and AtLPCAT2 in absence of LPC.** Incubations were performed as assays for the reverse reaction (see “Experimental Procedures”) but in the absence of added nonradioactive acyl-CoA during 30 min with 22 μg of yeast microsomal protein. The total amounts of radioactive acyl groups incorporated into PC per assay were for LPCAT1, 1.1 and 4.3 nmol for [^14^C]16:0-CoA and [^14^C]18:1-CoA substrates, respectively, and 2.8 and 5.4 nmol for LPCAT2, respectively. PC from six assays was pooled before lipase treatment, and the data presented were from duplicates of such pooled samples ± S.D.

The above data indicate that the LPCATs could have significant capacity to acylate the *sn*-1 position of LPC. We therefore investigated how much of the acyl groups from 16:0-CoA and 18:1-CoA was incorporated onto the different *sn* positions of PC in the absence of added LPC under conditions that promoted acyl exchange. Here, the incorporation at the different positions will largely be determined by the positional specificity of the rate-limiting step, *i.e.* the removal of acyl groups from PC in forming LPC, the substrate for the forward reaction. The proportion of label found at the *sn*-1 position ranged from 4.6 to 16.4%, and the proportion was significantly lower with 16:0-CoA than with 18:1-CoA substrates in assays with both AtLPCATs (Fig. 5).

In summary, it can be concluded from these experiments that LPCAT can both transfer and remove fatty acids to and from the *sn*-1 position of PC. The acylation and de-acylation rates of that position are substantially lower than for the *sn*-2 position under most assay conditions used, and 16:0 is strongly selected against in competition assays with 18:1 in acylation of either position.

##### Reverse Reaction of LPCAT with Ricinoleoyl-PC and Oleoyl-PC as Acyl Donors

**FIGURE 6.**
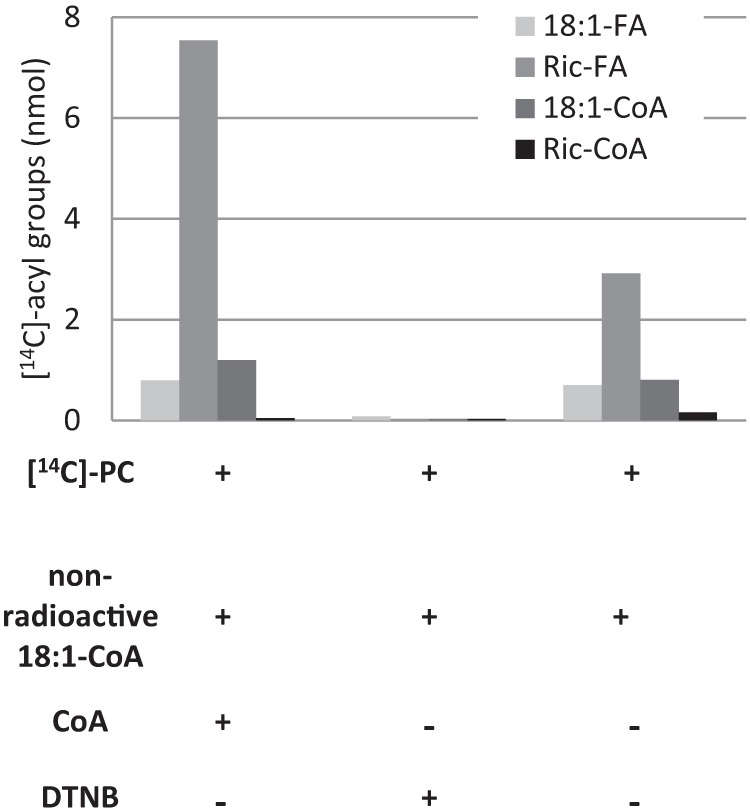
**Quantification of products of the reverse reactions of AtLPCAT2 with an equimolar mixture of *sn*-1–16:0-*sn*-2-[^14^C]18:1-PC and *sn*-1–16:0-*sn*-2-[^14^C]ricinoleoyl-PC.** Assays were performed as described for LPCAT reverse reaction conditions (see “Experimental Procedures”) with the additions and omissions indicated in the figure. DTNB was used at a concentration of 0.5 mm. Incubation time was 1 h. Four assays were pooled for extraction and separation of lipids. *Ric*, ricinoleoyl; *FA*, free fatty acids.

**FIGURE 7.**
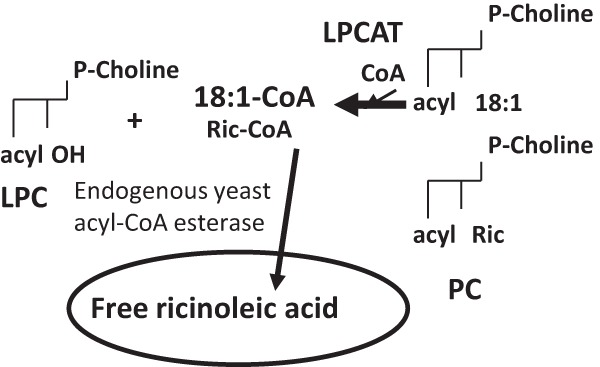
**Main reactions in the metabolism of the mixture of ricinoleoyl-PC and oleoyl-PC with expressed AtLPCAT in yeast microsomes.** Ricinoleoyl groups from PC are preferentially acylated to CoA over oleoyl groups to yield ricinoleoyl-CoA. The ricinoleoyl-CoA is then rapidly and preferentially hydrolyzed over oleoyl-CoA to free ricinoleic acid. The main products of LPCAT reversible reaction in these microsomes are thus oleoyl-CoA and free ricinoleic acid. *Ric*, ricinoleoyl.

We measured the reverse reaction of the AtLPCAT2 with an equimolar mixture of *sn*-2-[^14^C]18:1-PC and *sn*-2-[^14^C]ricinoleoyl-PC presented to the microsomal membranes under the conditions that promoted acyl exchange and trapped the formed radioactive acyl-CoA by adding large amounts of unlabeled 18:1-CoA. In this experiment, we also analyzed the ^14^C distribution in different lipid classes in the chloroform fraction after the assays. The acyl-CoA fraction was dominated by [^14^C]18:1 with only traces of [^14^C]ricinoleate found (Fig. 6). However, the chloroform fraction contained, unexpectedly, about four times more free [^14^C]ricinoleic acid than the sum of [^14^C]18:1 free fatty acid and [^14^C]18:1-CoA (Fig. 6). Omission of added free CoA lowered the amount of radioactivity found in free fatty acid and acyl-CoA by about 50% but did not change the ratio between radioactive oleic and ricinoleic acids in these lipid classes (Fig. 6). It should be noted that some free CoA, which is essential for the reverse reaction (Fig. 1*B*), was formed during these conditions due to the endogenous acyl-CoA thioesterase activity in the yeast membranes. To investigate if the formation of free ricinoleic acid was depending on the availability of free CoA, the assays were repeated with addition of DTNB. Only traces of radioactivity were found in free fatty acids and in the acyl-CoA fraction in the presence of this free CoA scavenger (Fig. 6). The results clearly showed that the formation of free ricinoleic acid was dependent on free CoA. Thus, the most likely explanation of the obtained results is that ricinoleoyl-CoA is formed by the reverse reaction of the LPCAT with free CoA and then rapidly and specifically hydrolyzed to free fatty acids after its formation. The main reactions in the metabolism of the mixture of ricinoleoyl-PC and oleoyl-PC with expressed LPCAT in yeast microsomes are summarized in Fig. 7.

##### Hydrolysis of Acyl-CoA

**FIGURE 8.**
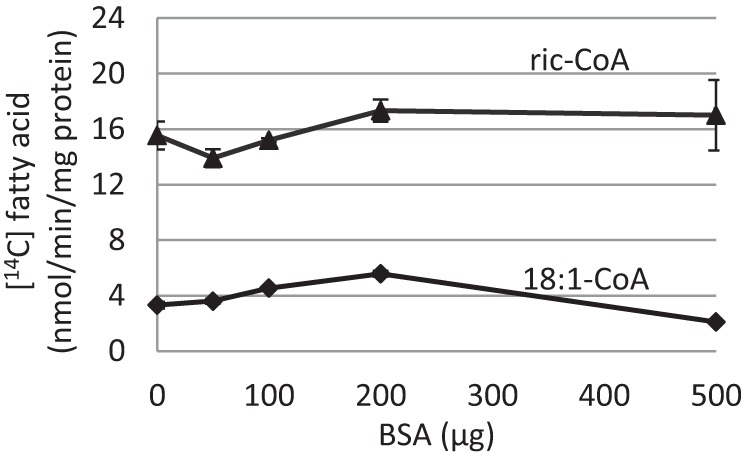
**Effect of BSA on yeast microsomal acyl-CoA esterase activity toward [^14^C]18:1-CoA and [^14^C]ricinoleoyl-CoA in microsomal preparations of yeast *ale1* microsomes transformed with empty vector.** Incubations contained 20 μg of microsomal proteins and 5 nmol of acyl-CoA and additions of BSA as indicated in the figure in a total volume of 100 μl. Incubation time for [^14^C]18:1-CoA was 10 min and for [^14^C]ricinoleoyl-CoA (*ric-CoA*) 2 min. Experiments were performed in duplicate ±S.D.

To investigate if this hydrolysis of ricinoleoyl-CoA was catalyzed by LPCAT or by endogenous yeast enzymes, microsomal preparations from yeast transformed with empty plasmid were incubated with 18:1-CoA and ricinoleoyl-CoA with increasing BSA concentrations. In the absence of BSA, the ricinoleoyl-CoA was hydrolyzed five times faster than 18:1-CoA (Fig. 8). The effect of BSA addition on the activity of the thioesterase with 18:1-CoA confirmed previous reports for acyl-CoA thioesterases (32). At low BSA concentration, the rate of hydrolysis increased, probably due to the disappearance of inhibitory acyl-CoA micelles. Higher BSA concentration caused a decrease in hydrolysis, presumably due to an inability of the esterase to operate on BSA-bound substrate. The hydrolysis rate of ricinoleoyl-CoA was, however, largely unaffected by addition of BSA, suggesting that this acyl-CoA may be unable to bind to BSA. It should be noted that even if the hydrolysis of ricinoleoyl-CoA was much higher than for 18:1-CoA, it was only about 3–15% of the acylation rate of ricinoleoyl-CoA to LPC (see *e.g.* Fig. 3*A*). Thus, the low acylation rate of ricinoleoyl-CoA to LPC compared with other unsaturated acyl-CoA substrates (Fig. 3) was not due to substrate depletion by a ricinoleoyl-CoA hydrolysis.

##### Acyl Specificities and Selectivities of LPCATs from Plants Accumulating Hydroxy Fatty Acids

In view of the above obtained results, indicating that LPCAT could be involved in removing ricinoleic acid from PC, we characterized yeast expressed LPCAT cDNA clones obtained from developing seeds of three species accumulating high amounts of hydroxy fatty acids in the seed oils. These enzymes were LPCATs from castor bean (*Ricinus communis*) and *Hiptage benghalensis,* both accumulating ricinoleic acid, and *Lesquerella fendleri* that accumulates lesquerolic acid ([11*Z*,14*R*]14-hydroxyicos-11-enoic acid). It should be noted that the first step in lesquerolic acid biosynthesis is the formation of ricinoleic acid on PC (33). In addition to these enzymes, we also characterized LPCAT from safflower (*Carthamus tinctorius*), a plant accumulating only “common” fatty acids in its seed oil.

**FIGURE 9.**
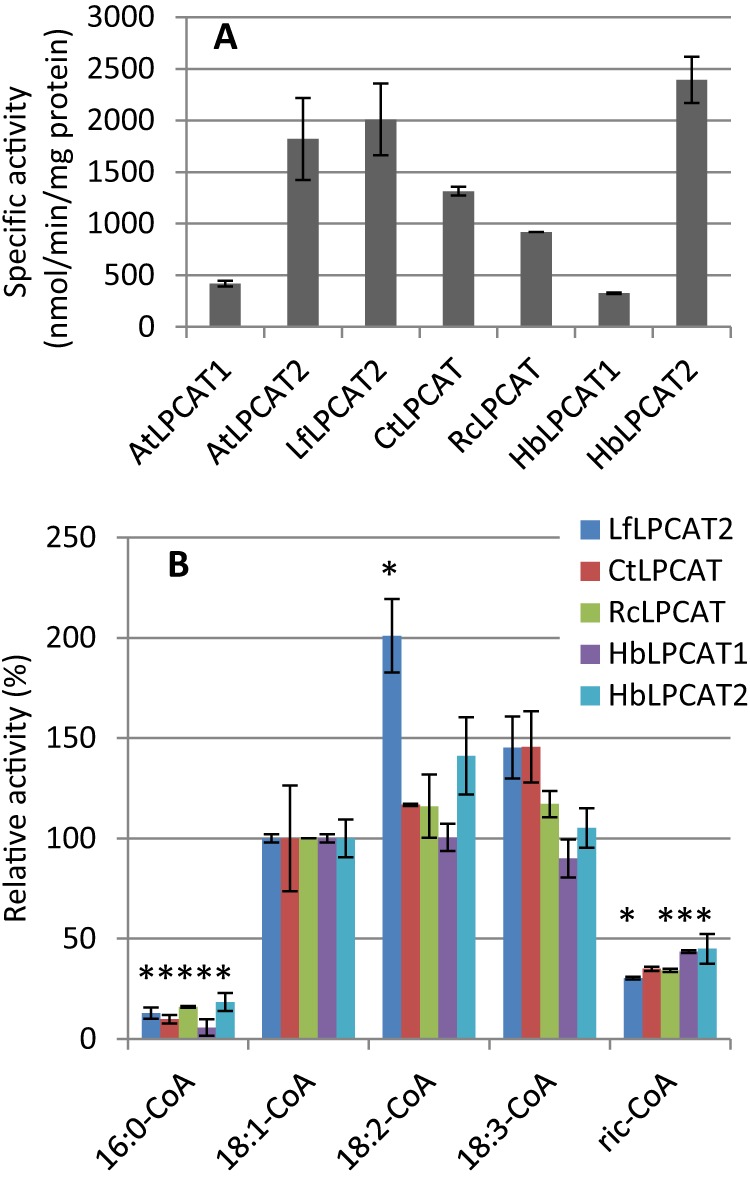
**Activities and specificities of various LPCATs in the forward reaction.** *A,* specific activity of various LPCATs using 18:1-CoA as an acyl donor. *B,* LPCAT activities for different acyl-CoA species relative to 18:1-CoA. Assays were performed as assays for the forward reaction of LPCATs using single acyl-CoA substrates (see “Experimental Procedures”). Results were from duplicate assays ±S.D. A significant difference (*t* test, *p* < 0.05) to 18:1-CoA is denoted with an *asterisk. Lf*, *L. fendleri*; *Ct*, *C. tinctorius*; *Rc*, *R. communis*; *Hb*, *H. benghalensis. Ric-CoA,* ricinoleoyl-CoA.

The specific activities in the forward reaction of the different LPCATs in microsomes from yeast expressing these enzymes varied between 200 and 2,300 nmol/min/mg of protein using 18:1-CoA as acyl donor, with LPCAT1 from *Arabidopsis* and *H. benghalensis* having the lowest specific activity (Fig. 9*A*). The acyl specificities for a range of acyl-CoAs were rather similar for all the C_18_-unsaturated acyl-CoAs except the acyl specificity of *L. fendleri* LPCAT2 that utilized 18:2-CoA nearly at a double rate in comparison with 18:1-CoA. Palmitoyl-CoA was very poorly utilized, whereas ricinoleoyl-CoA was a slightly better acyl donor for all LPCATs tested (Fig. 9*B*). We also expressed *LPCAT* genes from *Bernardia pulchella*, accumulating vernolic acid (12-epoxyoctadec-9-enoic acid), tung (*Aleurites fordii*), and bitter melon (*Momordica charantia*) that accumulate α-eleostearic acid (octadeca-9*Z*,11*E*,13*E*-trienoic acid) in their seed oils. However, the activities of these enzymes were more than 2 orders of magnitude lower than the activity of the other LPCATs, and for *Momordica* it was barely detectable (data not shown). Because of the low activity of these enzymes in the yeast microsomes, we were not able to get reproducible results for further characterization.

Our results on the acyl specificities of the castor bean LPCAT were in some respects in sharp contrast to those reported recently (34). The specific activities of the castor bean enzyme for 18:1-CoA, 16:0-CoA, and ricinoleoyl-CoA were reported to be in the same range as we report here, whereas 18:2-CoA was reported to be not utilized at all and 18:3-CoA very poorly. However, the authors of this paper have re-assayed the LPCATs with 18:1-CoA, 18:2-CoA, and 18:3-CoA and found that the published assays with 18:2 and 18:3-CoA were erroneous, and the activity with these substrates were similar to 18:1-CoA and thus very similar to our results.^4^

**FIGURE 10.**
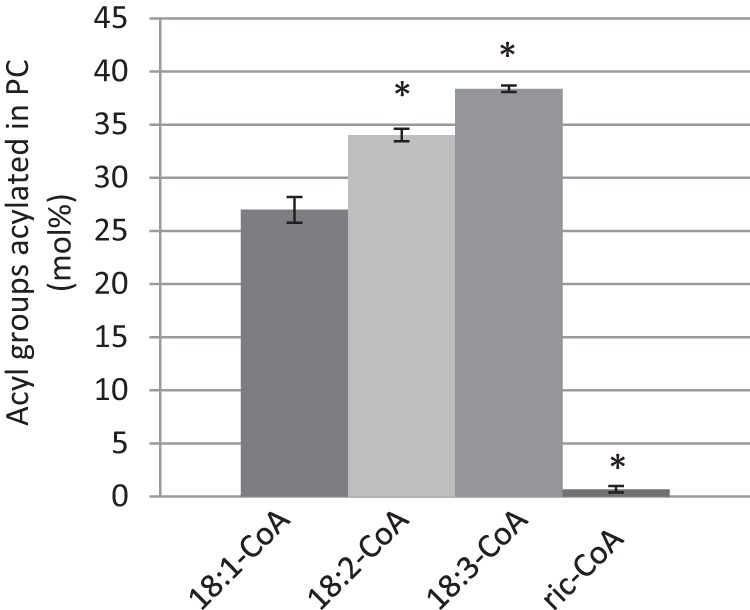
**Acyl substrate selectivities of castor bean LPCAT.** Acyl-CoA substrates (18:1-, 18:2-, 18:3-, and ricinoleoyl-CoA) were provided as an equimolar mixture with [^14^C]18:1-LPC. The data are from duplicate assays ± S.D. A significant difference (*t* test, *p* < 0.05) to 18:1-CoA is denoted with an *asterisk. Ric-CoA*, ricinoleoyl-CoA.

To further characterize the castor bean LPCAT, we performed the same acyl-CoA selectivity experiment as with *Arabidopsis* LPCATs (Fig. 10). The results showed that 18:1, 18:2, and 18:3-CoA were all well utilized, whereas ricinoleoyl-CoA was even more selected against than by the *Arabidopsis* LPCATs (compare Figs. 3*B* and 10).

**FIGURE 11.**
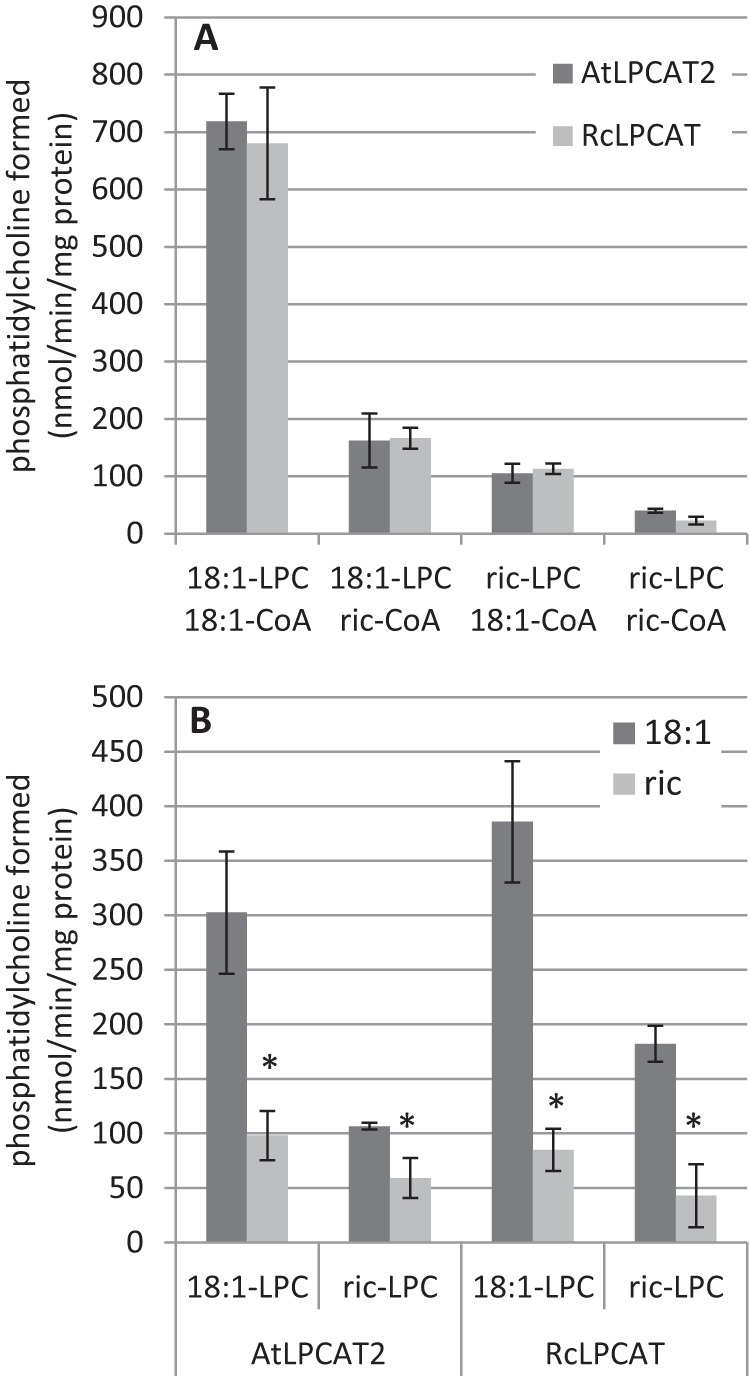
**Specificities and selectivities for ricinoleoyl and oleoyl containing acyl-CoA and LPC by AtLPCAT2 and castor bean LPCAT.** *A,* acyl-CoA provided as single molecular species. *B,* acyl-CoA provided as equimolar mixture of 18:1-CoA and ricinoleoyl-CoA. The data are from duplicate assays ± S.D. A significant difference (*t* test, *p* < 0.05) to 18:1-CoA is denoted with an *asterisk. Ric-LPC*, ricinoleoyl-LPC; *Ric-CoA*, ricinoleoyl-CoA.

The dominant TAG species in castor bean oil is tri-ricinoleoyl-TAG, and thus, the last step in formation of TAG must primarily use di-ricinoleoyl-DAG. Because ricinoleic acid is produced on PC, it can be speculated that di-ricinoleoyl-PC is formed in the seed and rapidly converted to di-ricinoleoyl-DAG, to be utilized in TAG synthesis. We therefore tested how efficient castor bean LPCAT utilized ricinoleoyl-LPC in comparison with 18:1-LPC in acylation of either oleoyl-CoA or ricinoleoyl-CoA or a mixture of these acyl-CoA species. We also compared the results with the corresponding incubations with AtLPCAT2. Both enzymes showed the same specificities when single acyl-CoA substrates were presented with ricinoleoyl-LPC with ricinoleoyl-CoA being a much poorer substrate than the 18:1-containing substrates (Fig. 11*A*). The combination of ricinoleoyl-LPC and ricinoleoyl-CoA gave the lowest acylation rates (Fig. 11*A*). We then presented the enzymes an equimolar mixture of 18:1-CoA and ricinoleoyl-CoA together with either 18:1-LPC or ricinoleoyl-LPC (Fig. 11*B*). The proportion of ricinoleoyl groups acylated was always less than oleoyl groups in all incubations. However, ricinoleoyl-CoA groups competed much better with 18:1-CoA compared with the competition assays done also with 18:2-CoA and 18:3-CoA included (Fig. 9). The highest ratio of ricinoleoyl to oleoyl groups acylated was about 0.5 and was obtained with AtLPCAT2 with ricinoleoyl-LPC. It can be concluded that castor bean LPCAT did not preferentially produce di-ricinoleoyl-PC, and thus the di-ricinoleoyl-DAG used for TAG synthesis in castor bean is unlikely to be derived from re-editing of PC by LPCAT.

**FIGURE 12.**
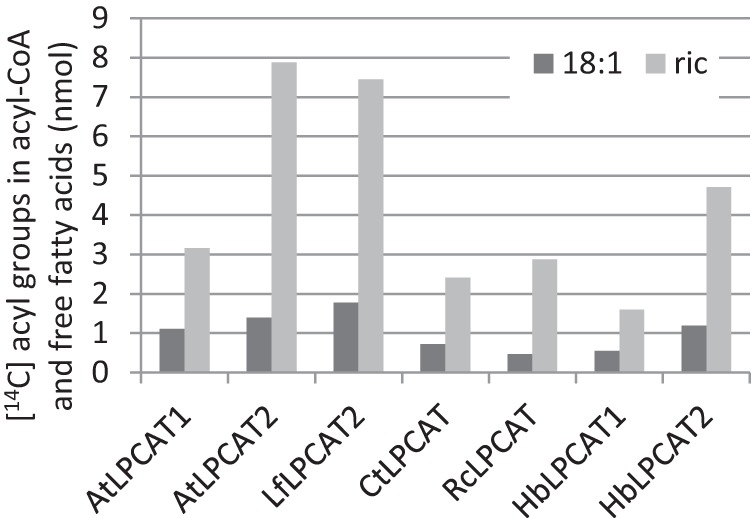
**Quantification of radioactive products of the reverse reactions of various LPCATs using with an equimolar mixture of *sn*-1–16:0-*sn*-2-[^14^C]18:1-PC and *sn*-1–16:0-*sn*-2-[^14^C]ricinoleoyl-PC as substrates.** Each *bar* represents the sum of radioactivity found in the acyl-CoA and free fatty acids of the particular acyl group. In the absence of acyl exchange, only trace amounts of radioactivity were found in any of these fractions (see Fig. 6, assays with DTNB). Assays were performed as described under “Experimental Procedures” for the reverse reaction of LPCAT with added nonradioactive 18:1-CoA for 1 h. Four assays for reverse reaction were pooled before extraction and separation of lipids. *Dark gray bars*, [^14^C]18:1-CoA and free acid; *light gray bars* [^14^C]ricinoleoyl-CoA and free acid. *Lf*, *L. fendleri*; *Ct*, *C. tinctorius*; *Rc*, *R. communis*; *Hb*, *H. benghalensis*; *Ric*, ricinoleoyl.

We assayed the selectivity in the reverse reaction of the various LPCAT enzymes with an equimolar mixture of *sn*-2-[^14^C]18:1-PC and *sn*-2-[^14^C]ricinoleoyl-PC and added unlabeled 18:1-CoA and compared it with the *Arabidopsis* enzymes (Fig. 12). All seven LPCATs catalyzed the removal of ricinoleoyl groups from PC faster than 18:1, with a ratio ranging from 3-fold for *H. benghalensis* LPCAT1 to 6-fold for castor LPCAT and *Arabidopsis* LPCAT2. As in the corresponding previous incubations with *Arabidopsis* LPCAT2 (Fig. 6), nearly all of the removed ricinoleoyl groups were found as free ricinoleic acid in all assays (data not shown).

## DISCUSSION

PC is not only the major membrane phospholipid in plant cells but also the substrate for production of polyunsaturated fatty acids and a number of unusual fatty acids produced by desaturase-like enzymes (1). Therefore, the precursor fatty acid for all these fatty acids has to be efficiently channeled into PC and the modified acyl groups transferred out of PC to other lipids, including into TAGs in oil seeds, where they can occur in very high amounts. Two enzyme systems have recently been shown to be involved in this channeling of fatty acids into PC and out of PC in *Arabidopsis* seeds, the PDCT and the LPCATs (35). The PDCT converts DAG produced by the Kennedy pathway to PC by catalyzing the transfer of a phosphocholine group directly from PC to DAG and thereby equilibrates the DAG moieties of these two molecules. An *Arabidopsis* insertion mutant of the PDCT coding gene (*ROD1*) reduces the amount of polyunsaturated fatty acids in seed TAGs by 40% (12). Although a double knock-out of *Arabidopsis lpcat1/lpcat2* only slightly reduced the polyunsaturated fatty acids in seed TAGs (36), a triple insertion mutant (*rod1/lpcat1/lpcat2*) reduced the content of these fatty acids in TAG by 66% (35). These experiments clearly show that LPCATs and PDCT, working in concert, are the main enzymes responsible for the channeling of PC-derived fatty acids into TAG in developing *Arabidopsis* seeds.

During [^14^C]acetate labeling of developing seeds of soybean and *Arabidopsis*, 86 and 70%, respectively, of the labeled fatty acids initially incorporated into PC were confined to the *sn*-2-position (36, 37). In contrast, an *Arabidopsis* double mutant of *lpcat1/lpcat2* showed a nearly equal distribution of the label in both *sn* positions of PC, even at short incubation times, mirroring the distribution of label in DAG (35, 36). This strongly suggests that LPCAT enzymes are responsible for the immediate incorporation of acyl groups exported from the plastid into PC at the *sn*-2 position of PC. The results presented here indicate that LPCAT1 and LPCAT2 can catalyze both the acylation and de-acylation of fatty acids at the *sn*-1 position of PC. The rate of acylation at *sn*-1 ranged from 15 to 70% that of the *sn*-2 position in assays with ether analogs of LPC, and the positional preference was highly dependent on both acyl-CoA concentration and acyl-CoA species. These *in vitro* data are consistent with the hypothesis that the initial incorporation of 18:1 at the *sn*-1 position of PC *in vivo* (37) also could be carried out by LPCAT. In developing soybean embryos, the ^14^C-labeled acyl groups at the *sn*-1 position of PC contained even at short labeling times about 25% of saturated acyl groups, suggesting also that saturated fatty acids could be directly incorporated onto the *sn*-1 position of PC (37). The acylation of *sn*-1 and *sn*-2 ether LPC analog by *Arabidopsis* LPCAT1 and LPCAT2 showed that 16:0 is strongly selected against compared with 18:1 in acylation of both *sn* positions under all different assay conditions used. Therefore, the presence of 16:0 at the *sn*-1 position of PC in *Arabidopsis* is not likely to involve LPCATs to any significant extent, but rather it may be due to incorporation from newly synthesized DAG, either via PDCT or CDP-choline:DAG phosphotransferase. The strong selection against 16:0 in acylation in either the *sn*-1 or *sn*-2 position of PC indicates that PC acyl remodeling catalyzed by LPCAT reduces the amount 16:0 in PC and thereby reduces 16:0 content in PC-derived DAG compared with *de novo* synthesized DAG.

Seven different LPCAT enzymes derived from five different plant species were characterized, and all showed similar acyl specificities. Acyl-CoA derivatives of 16:0 and ricinoleic acid were poorly utilized and were almost outcompeted by unsaturated acyl-CoAs, whereas unsaturated C_18_ acyl groups were all well utilized. This indicates that ricinoleic acid is likely not entering PC through the action of LPCAT enzymes. In plants accumulating ricinoleic acid-rich oil, this fatty acid is synthesized by hydroxylation of 18:1 esterified mainly at the *sn*-2-position of PC and then specifically removed for its subsequent channeling into TAG (6). It can therefore be assumed that once transferred to the acyl-CoA pool, ricinoleoyl groups will not re-enter PC by the action of LPCATs.

The LPC substrate for acylation catalyzed by LPCAT could be generated by the action of phospholipase A on PC. This hypothesis was put forward by Lands (5) as the mechanism for acyl remodeling of PC, the so-called Land's cycle. Another hypothesis was that LPCAT itself generates its own LPC acyl acceptor by its reverse reaction (16). The latter hypothesis has attractive features because it does not necessitate a lipase and an activation of the liberated fatty acid to acyl-CoA, and thus, instead of highly coordinated actions of three enzymes, it only needs one enzyme and no ATP. Overexpression of LPCAT1 and LPCAT2 in *Arabidopsis* showed a significant increase in polyunsaturated fatty acids in seed oil (36). Because the removal of fatty acids from PC is likely to be the rate-limiting step in the acyl editing by LPCAT, this effect cannot easily be explained by the removal of polyunsaturated fatty acids catalyzed by phospholipases. Instead, it supports the hypothesis that the transfer of the fatty acids formed on PC to the acyl-CoA pool and further to TAG is at least to some extent catalyzed by the reverse reaction of LPCAT and further channeled to TAGs.

Our results clearly show that the LPCATs in microsomal preparations can carry out the reverse reaction at rates corresponding to what can be measured in forward reaction for other microsomal acyltransferases expressed in yeast. In the reverse reaction, the AtLPCAT enzymes did not discriminate between any of the unsaturated C_18_ acyl groups and had a high selectivity for the *sn*-2 position. The results further imply that there is a direct transfer of acyl groups from PC to free CoA with no free fatty acid intermediate formed, because free CoA was essential for the reverse reaction. In order for the reverse reaction to be significant *in vivo*, the enzyme activity has to be high and the concentrations of LPC and acyl-CoA very low compared with the concentrations of PC and free CoA. These three criteria are likely to be valid *in vivo*. The specific activity of the *Arabidopsis* LPCAT2 in the forward reaction, when expressed in yeast microsomes, was about 100 times higher than other acyl-CoA acyltransferase activities that have been expressed in such microsomes (26, 27). LPC and PC concentrations in *Arabidopsis* seed cells have been reported to be about 0.05% (36) and 48% (38), respectively, of all polar lipids. The bulk of the acyl-CoAs is believed to be bound to ACBP in cells and might therefore not be available for the enzyme. The concentration of free acyl-CoA in cells has been estimated to be in the nanomolar range (39). We could not find any reports on levels of free CoA in the cytosol of plant cells, but in animal cells they have been reported to be around 5 μm, *i.e.* 3 orders of magnitude higher than the free acyl-CoA concentration (40). It should be noted that the driving force behind the sustained reverse reaction of LPCAT is the reacylation of the formed LPC by the forward reaction of the same enzyme that will keep the LPC concentration low and result in an equilibration between the acyl groups in the acyl-CoA pool and acyl groups in PC.

Castor bean PDAT was suggested to specifically transfer ricinoleoyl groups from PC to TAG (21). Co-expression of castor bean PDAT together with castor bean Δ12-hydroxylase led to a decrease of ricinoleoyl groups in PC and an increase in TAG concomitant with a restoration of oil content in the seeds to near wild-type levels (31). This strongly supports the idea that castor bean PDAT plays a significant role in channeling ricinoleoyl groups from PC to TAG. The specific removal of ricinoleic acid from PC by phospholipases with subsequent activation to acyl-CoA and further channeling into TAG has also been proposed (6, 41). However, no candidate genes for such phospholipases have yet been identified. LPCAT has previously not been suggested to be involved in the selective removal of ricinoleoyl groups from PC. Very unexpectedly, we found that ricinoleoyl groups in PC were removed up to six times faster than 18:1 by the reverse reaction of AtLPCAT2, despite that they were very poor substrates in the forward reaction. A selectivity for ricinoleoyl groups was seen for castor bean, *H. benghalensis,* and *L. fendleri* LPCATs, plants that naturally accumulate TAG rich in hydroxy fatty acids, but also for LPCATs from safflower, a plant that, like *Arabidopsis*, does not accumulate such fatty acids. Thus, LPCAT is a potential candidate enzyme for the specific transfer of ricinoleoyl groups from PC directly to the acyl-CoA pool by its reverse reaction. Because *Arabidopsis* LPCAT2 was as efficient and selective as the castor bean enzyme, it implies that transfer of ricinoleoyl groups from PC to the acyl-CoA pool should not constitute any bottleneck for the accumulation of high proportions of ricinoleic acid in the seed oil of *Arabidopsis* expressing the castor bean Δ12-hydroxylase. *In vivo* radioactive labeling experiments in transgenic *Arabidopsis* strongly support this suggestion by showing that there is a highly efficient mechanism for transferring ricinoleoyl groups from PC to newly synthesized DAG in these seeds. In labeling experiments with developing *Arabidopsis* seeds expressing the castor bean Δ12-hydroxylase, less than 2.5% of the fatty acids found in PC were hydroxylated, but about half of the newly synthesized DAG species contained a hydroxylated fatty acid (42). This indicates that the acyl-CoA pool that is utilized by the *sn*-glycerol 3-phosphate and lysophosphatidic acid acyltransferases in these developing seeds is highly enriched in ricinoleoyl groups compared with the concentration of this fatty acid in PC. The bottleneck in the accumulation of hydroxylated fatty acids in the seed TAG in developing *Arabidopsis* appeared instead to be in the utilization of ricinoleoyl containing DAG (42). This bottleneck could be alleviated by co-expression of castor bean DGAT and PDAT (31). All LPCATs tested in this study showed a higher rate of removal of ricinoleic acid than of 18:1 from PC. The ratio of ricinoleyl to 18:1 removal varied from 3 to 6, which may suggest that this specificity is not necessarily an intrinsic property of all LPCAT enzymes. Because microsomal fractions from developing seeds that are not producing ricinoleic acid showed high and specific phospholipase activities toward ricinoleoyl-PC, it was suggested that these enzymes might serve as a general scavenger for oxygenated fatty acids in the membranes, protecting them from damage (41). The results here presented indicate that the reverse reaction catalyzed by LPCAT also might serve this purpose.

Like all microsomal membranes, the yeast membranes contain acyl-CoA esterases. We showed that these esterases have much higher activity with ricinoleoyl-CoA than with oleoyl-CoA and resulted in a near complete hydrolysis of ricinoleoyl-CoA formed by the reverse reaction catalyzed by LPCAT. Furthermore, the rate of hydrolysis was essentially unaffected by addition of BSA, whereas an excess amount of this protein caused a significant decrease in hydrolysis of oleoyl-CoA. Although this was not investigated further, it may suggest that ricinoleoyl-CoA does not bind to BSA. It is believed that most acyl-CoA in the cell is bound to ACBPs (39). It has been shown that *Arabidopsis* ACBP and BSA have similar and strongly inhibitory effects on acyl-CoA hydrolysis by safflower microsomal acyl-CoA esterases (43). As far as we know, there are no reports on the binding capacity of ricinoleoyl-CoA and other oxygenated acyl-CoAs to ACBPs. However, it can be speculated that high intrinsic activity of acyl-CoA esterases toward oxygenated acyl-CoAs, possibly combined with an inability of ACBP proteins to protect them from hydrolysis, prevents these acyl groups from being used by acyltransferases in the formation of membrane lipids that could affect membrane integrity and function. Thus, it is possible that specialized ACBPs in castor bean seeds and other plants that accumulate high amounts of oxygenated fatty acids in their seed oils might play a role in maintaining them in the acyl-CoA pool for their further channeling by acyltransferases into the oil.

In conclusion, published *in vivo* labeling studies of developing seeds of *Arabidopsis* and soybean suggest that a majority of the fatty acids newly exported from the plastid is not first acylated to the glycerol backbone via the Kennedy pathway but is instead entering PC, mainly at the *sn*-2 position (36, 37). Studies of *Arabidopsis* disrupted in *LPCAT* genes suggest that LPCATs are responsible for this initial incorporation into PC (35, 36). Our biochemical studies suggest that LPCATs can incorporate acyl groups at both *sn* positions and thus could be involved in direct incorporation of acyl groups also at the *sn*-1 position of PC. Furthermore, we showed that LPCAT is responsible for the *in vitro* acyl exchange between acyl-CoA and PC, as suggested nearly 30 years ago using microsomal fractions from developing safflower seeds (16). We also consider that *in vivo* conditions could support such a reverse reaction at rates that are physiologically significant. We further showed that ricinoleoyl groups are preferentially and rapidly removed in the reverse reaction of LPCATs but poorly utilized in the forward reaction. Published *in vivo* studies are consistent with our *in vitro* studies suggesting an important role for LPCATs in transferring polyunsaturated and ricinoleoyl groups from PC directly into the acyl-CoA pool by acyl exchange.

It is perhaps impossible by traditional *in vivo* radioactive labeling experiments to conclusively determine whether acyl exchange between PC and acyl-CoA is occurring via the reverse reaction of LPCAT or by the release of fatty acids mediated by phospholipase A and the subsequent activation to acyl-CoA. An approach using [^13^C_2_,^18^O_2_]acetic acid isotope labeling of developing *Arabidopsis* seeds knocked out in their PDAT and PDCT activities is likely to yield information to which extent the fatty acids formed on PC are passing through a free fatty acid intermediate before ending up in TAGs. Such an approach has previously been used successfully to establish that acyl groups formed *de novo* in the plastid pass through a free fatty acid intermediate before being activated to acyl-CoA and utilized in cytosolic lipid synthesis (44).

Our findings of very high activities of yeast acyl-CoA esterases toward ricinoleoyl-CoA and the inability of BSA to inhibit this hydrolysis warrants future studies on specificities of plant acyl-CoA esterases toward oxygenated fatty acids and the abilities of plant ACBP proteins to bind such acyl-CoA species.

## Footnotes

4 F. Maroto, personal communication.

## Footnotes

3 The abbreviations used are:

PC: phosphatidylcholine
LPCAT: lysophosphatidylcholine acyltransferase
16:0: palmitic acid
18:1: oleic acid
TAG: triacylglycerol
18:2: linoleic acid
18:3: α-linolenic acid
LPC: lysophosphatidylcholine
DAG: diacylglycerol
PDCT: phosphatidylcholine:diacylglycerol cholinephosphotransferase
PDAT: phospholipid:diacylglycerol acyltransferase
OGPC: *O*-9-*cis*-octadecenyl-*sn*-glycero-3-phosphocholine
DTNB: dithionitrobenzoic acid
ACBP: acyl-CoA binding protein.
